# Vasectomy: A Long, Slow Haul to Successful Takeoff

**DOI:** 10.9745/GHSP-D-16-00355

**Published:** 2016-12-23

**Authors:** James D Shelton, Roy Jacobstein

**Affiliations:** a Editor-in-Chief, Global Health: Science and Practice, Washington, DC, USA.; b Senior Medical Advisor, IntraHealth International, Chapel Hill, NC, USA.

## Abstract

Vasectomy use is plagued by low demand among men. Nevertheless, its compelling advantages make substantial investment worthwhile. On the supply side, a priority is to actively link vasectomy with service delivery approaches for the other highly effective long-acting and permanent clinical methods. Robust demand generation should include messaging specific to vasectomy, but should also draw on broader social and behavior change communication efforts increasingly aimed at engaging men in family planning.

Despite vasectomy's well-recognized benefits including high contraceptive effectiveness, convenience, permanence, relative ease of provision, few side effects, and high levels of satisfaction, use of the method has plateaued globally (Figure)^1^^–^^3^ and continues to languish in most low- and middle-income countries, including having a 0.0% prevalence in Africa.^4^ This issue of GHSP includes a review by Shattuck et al. of program reports and research on vasectomy, in which the authors also advocate increased support for vasectomy.^5^ The review has some gaps, in part because of limitations of the review criteria. Nevertheless, we publish the article because we believe it is important to share such evidence as widely as possible, particularly since vasectomy is one of only two modern male contraceptive methods available (along with condoms). Moreover, we provide our own additional perspective here because we believe vasectomy merits more attention and advocacy—recognizing that fulfilling the potential for vasectomy will require long-term and substantial investment.

**FIGURE:**
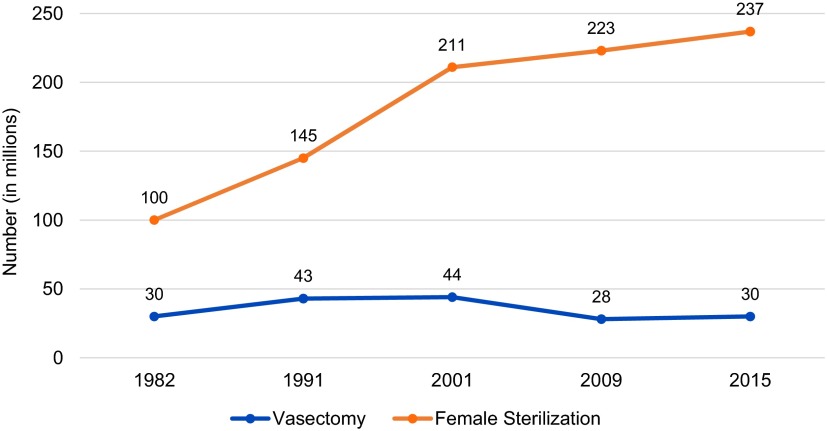
Trends in Worldwide Use of Permanent Contraceptive Methods Estimates based on UN 2015,^1^ UN 2012,^2^ and EngenderHealth 2002.^3^

## LOW DEMAND FROM MEN IS THE OVERRIDING ISSUE

Let's put front and center the fundamental underlying constraint to vasectomy uptake—low demand for the method among men in low- and middle-income countries. Among the many reasons for low demand:

- First and foremost, men and women typically see contraception as women's responsibility, partly because women bear the brunt of childbearing—thus a gender-equality issue.
- Simple awareness of vasectomy is the lowest, by far, among all highly effective methods.
- Even when men and women know of the method, their knowledge is fraught with myths and misconceptions—notably that vasectomy is castration or makes men weak.
- Couples often do not discuss any kind of contraception, including vasectomy, among themselves.
- Men are squeamish about physical contact with the area of the scrotum and testes.
- Men seek routine health care less frequently than women and have little familiarity with the health system.
- Providers themselves often have poor knowledge about vasectomy or bias against it, and so they fail to discuss it or provide accurate information to clients.
- Deciding to have a vasectomy requires coming to a psychological resolution that one's reproductive years have come to an end.
- Getting a vasectomy is a new and one-time act with which men have no familiarity and thus lack self-efficacy.
- As with adoption of any method of contraception, potential clients may sometimes have many other priorities in their lives that take precedence.

## THOUGHTS ON THE REVIEW

We appreciate the contribution Shattuck and colleagues have made in assembling their review, including reaching deeply into the gray literature, framing the findings according to the Supply–Enabling Environment–Demand (SEED) model, and providing productive insights. And the authors do address the pivotal demand conundrum. Still, we would have preferred if they had taken on more fully the overriding issue of weak demand, laying out its complete scope and challenges. Another concern is that their analysis largely lacks program outcome results. The reality is that vasectomy programming has generally been carried out through modestly resourced pilot programs of short duration—yielding very modest results. We need to acknowledge that reality.

Also, since the overriding problem is very low demand, supply-side issues such as task shifting, training, vasectomy technique, and mobile outreach that were covered in the review have some relevance but are still rather secondary. Focusing on them can detract from attention to the main issue of limited demand. Moreover, focusing on those supply-side issues can foster the misapprehension that if only we could make vasectomy more accessible, its use would rise substantially.

Lastly, we see little merit in the article's proposal of active integration of vasectomy with current male circumcision programming. The large majority of male circumcision recipients for HIV prevention currently are very young men—even boys. Conversely, the main audience for vasectomy is much older men who are interested in having no additional children. Likewise, male circumcision providers, particularly those working on programs offering voluntary medical male circumcision for HIV prevention, are often fully occupied with providing male circumcision and may have little knowledge of family planning provision. Merely training them in vasectomy, especially in the nearly universal context of low vasectomy demand, doesn't seem very worthwhile. We do see value, however, in trying to reach these boys and young men with messages on contraception and reproductive health in general and on positive gender norms.

## WHY INVEST IN VASECTOMY?

It is reasonable to ask how much investment in vasectomy currently makes sense, compared with alternative investments, recognizing that resources are limited. For example, long-acting reversible contraceptives (LARCs), particularly implants, have many of the same positive attributes as vasectomy, are in high demand, and are being effectively provided at very large scale.^6^^,^^7^ Nevertheless, in our view, the following points argue for increased attention to vasectomy:

- Men's and women's fertility preferences are generally now comparable in many countries.^8^^,^^9^
- Demand for effective modern contraception in general will continue to rise.
- Demand for limiting further births is already very high, exceeding demand for spacing among married women in most regions of the world and rising in Africa.^10^^,^^11^
- Female sterilization is the most widely used contraceptive method in the world—more than 235 million women rely on it (Figure), and it has substantial use even in some very low-income African countries via mobile services.^12^
- Vasectomy has the many positive method characteristics we noted above, and is easier and safer to provide than female sterilization.
- Social norms on gender equality are changing in a positive direction and that change will probably accelerate.
- Supporting wider individual and couple choice promotes better client satisfaction and use of contraception, as well as individual rights.

Notably, evidence from a number of countries demonstrates that over time vasectomy can account for a significant component of contraceptive use. It comprises 24%–31% of such use in some countries with high socioeconomic development, such as Canada, New Zealand, South Korea, and the United Kingdom, and it also has sizable use in several low- and middle-income countries, including Brazil, Bhutan, Iran, and Nepal.^4^

## GETTING BEYOND THE SMALL PILOT PARADIGM BY PACKAGING VASECTOMY WITH OTHER CLINICAL METHODS

Clearly, getting to a vasectomy takeoff requires emphasis on good-quality services and wide access on the supply side as well as a robust demand component. Heretofore, the typical approach to vasectomy has been to nurture a selected number of dedicated champion providers, intended to become a hub of expanded programmatic activity. But these efforts have been small and limited in funding, scope, duration, and priority. Establishing a nucleus of committed, well-supported vasectomy providers who can serve as champions for the method and a platform for expansion continues to make sense. This model appears to be beginning to take hold in Rwanda, which (albeit an exceptional country for health service delivery) has 0.2% contraceptive prevalence for vasectomy (2014-15 DHS), compared to 0.0% in 2010.^13^^,^^14^

But linking vasectomy more squarely to existing service delivery platforms such as mobile outreach, which is currently providing widespread, high-quality access to LARCs and female sterilization, offers another major opportunity. We have already seen that making intrauterine devices (IUDs) available in the context of high-quality provision of implants improves use of IUDs—which have long tended to be underutilized.^15^

## HARMONIZING VASECTOMY DEMAND GENERATION WITH BROADER FAMILY PLANNING COMMUNICATION AIMED AT MEN

Since demand is clearly the overriding constraint, intensive and sustained demand generation must be a key part of the solution. Some of that demand support, of course, needs to be specifically about vasectomy including promoting the benefits of vasectomy and dispelling misconceptions about it. But men are increasingly the target audience of social and behavior change communication efforts for family planning more generally. Given limited resources, the effort to increase vasectomy demand should draw on harmonized broader family planning demand support aimed at men. Examples of this broader messaging include:

- Promoting a positive image for family planning
- Increasing couple communication
- Advancing the advantages of healthy timing and spacing of pregnancies, including limiting fertility for those who have reached desired family size
- Promoting an active role for men in pregnancy planning
- Projecting images such as the “permanent smile” of vasectomy users^16^
- Projecting the potential better sexual satisfaction when the couple is freed from the worry of unwanted pregnancy

Moreover, broader development efforts to advance gender equality should, in turn, promote the appropriate role for men in family planning as a client, supportive partner, and advocate.

## CONCLUSION

We believe serious, increased, and sustained support to vasectomy is warranted. But no one should harbor any illusion that substantial impact will occur quickly. Rather, it calls for plugging away, year after year, until takeoff is reached and a substantial proportion of men in low- and middle-income countries opt for vasectomy.

## Notes

**First Published Online:** 2016 Dec 23
